# Prevalence and profiles of adverse childhood experiences: a French nationwide study using the CONSTANCES cohort

**DOI:** 10.1136/bmjph-2025-003300

**Published:** 2026-04-09

**Authors:** Aicha Mourchidi, Fabienne El-Khoury, Maria Melchior, Marie Zins, Emmanuel Wiernik, Judith van der Waerden

**Affiliations:** 1 Social Epidemiology, Mental Health and Addictions team, Institut Pierre Louis d’Epidemiologie et de Sante Publique, Sorbonne Université, INSERM, Paris, France; 2 Population-Based Cohorts Unit INSERM UMS011, Université Paris Cité, Paris Saclay University, Université de Versailles Saint-Quentin-en-Yvelines, Paris, France

**Keywords:** Epidemiology, Public Health, Sex Factors, Prevalence

## Abstract

**Introduction:**

Adverse childhood experiences (ACEs) can impact individuals’ health later in life. In France, research on the prevalence and patterns of ACEs remains limited. This study aims to estimate the prevalence of different categories and profiles of ACEs in the French general population, and to explore their associations with childhood socioeconomic and demographic factors.

**Methods:**

A cross-sectional observational study was conducted using retrospective data from the CONSTANCES cohort, a large population-based cohort study in France, including adults aged 18 to 69 years at enrolment. The analytic sample included 101 071 participants with complete data on ACEs. Prevalence and typology of ACEs was assessed using the Behavioural Risk Factor Surveillance System questionnaire and examined individually, cumulatively and through latent class analysis (LCA) to identify exposure profiles. Associations with socioeconomic and demographic factors were examined using multinomial logistic regression, and all analyses were stratified by sex.

**Results:**

Overall, 65% of participants reported experiencing at least one ACE. Females were more likely than males to report certain experiences, including sexual violence. LCA identified three distinct ACE profiles in males (low exposure; physical and emotional violence; unstable family environment), and four in females (similar to males, with an additional class characterised by sexual violence). Class membership was associated with factors such as age, parental socioprofessional category and geographical origin.

**Conclusions:**

ACEs are highly prevalent in the French population and differ by sex. These findings emphasise the importance of social determinants, including gender and socioeconomic position, as risk factors.

**WHAT IS ALREADY KNOWN ON THIS TOPIC**

- Adverse childhood experiences (ACEs) are common and strongly associated with long-term physical and mental health issues, with notable differences in exposure patterns between women and men.
- In France, data on the prevalence and clustering of ACEs are scarce; representative studies using more detailed, sex-sensitive analytical approaches are needed to address this gap.

**WHAT THIS STUDY ADDS**

- Using data from over 100 000 adults in a large, nationally representative French cohort, this study shows that ACEs are highly prevalent, reported by 70% of females and 67% of males.

**HOW THIS STUDY MIGHT AFFECT RESEARCH, PRACTICE OR POLICY**

- The identification of distinct ACE exposure profiles through latent class analysis offers new insight into how childhood adversity clusters in the French population and varies according to age, parental occupation and geographical origin.

## Introduction

 Adverse childhood experiences (ACEs) are potentially traumatic events that cause intense stress and can have lasting effects on an individual’s physical, emotional and psychological well-being over their lifespan. In one of the seminal studies on this topic, ACEs were defined across different categories including forms of abuse, neglect, as well as household dysfunction.^1^ Initially conceptualised in studies in the USA, the scope of ACEs has since broadened and is now used globally to encompass a wide range of adversities.^1 2^ These experiences are strongly linked to long-term physical and mental health outcomes, including chronic disease, psychological disorders and engagement in risky health behaviours.^3^,^5^ ACEs are recognised by the WHO as a major public health concern due to their lifelong impact and contribution to premature mortality.^6 7^ Research across diverse settings consistently demonstrates their high prevalence: 61% of adults in the USA, 43% in Germany and over 70% in several African countries report at least one ACE.^7^,^9^ Although prevalence rates vary across countries, certain consistent findings emerge, notably sex differences in both the frequency and type of ACE exposure. Women are more likely than men to report a higher number of adversities and to disproportionately encounter specific types of ACEs, such as exposure to sexual violence. However, to elucidate the underlying mechanisms of these sex differences, additional evidence from sex-specific investigations is needed.^10 11^ ACE prevalence also varies across global contexts, particularly in socioeconomically fragile regions. For instance, a recent study conducted in Cité Soleil, Haiti, reported that 100% of participants experienced at least one ACE, with multidimensional adversity shaped by poverty, community violence and limited access to services. Such findings highlight the influence of socioeconomic and demographic conditions on childhood adversity and underscore the importance of generating context-specific data.^12^

In France, comprehensive and detailed data on ACEs remain limited. Most existing studies are based on small samples or selected populations^13 14^ and tend to focus on specific health outcomes such as eating disorders, repeated abortion or muscle strength, without providing comprehensive estimates of ACE prevalence.^15^,^17^ To inform effective public health actions, robust data from large, more representative samples are essential. In addition to broader sampling, methodological improvements are also needed to more accurately capture the complexity of ACE exposure. Most studies rely on the cumulative risk approach, which sums the number of adversities experienced by an individual to produce a total ACE score.^18 19^ While this method offers simplicity and ease of use, it assumes that all adversities carry equal weight and fails to capture patterns of co-occurrence between different adversities. To address some of these limitations, latent class analysis (LCA) has gained traction as a method that can identify distinct subgroups of individuals with similar ACE patterns, offering a more nuanced understanding of how adversities cluster and vary across populations.^20^

The aim of this study is to assess the prevalence and distribution of ACEs in the French adult population, using data from a large general population cohort. First, we describe the different types of ACEs reported and estimate their sex-specific prevalence, employing three complementary approaches: analysis of each type of adversity separately, the cumulative risk approach and LCA. Second, rather than focusing on the long-term consequences of ACEs, we examine the socioeconomic and demographic family contexts in which participants were raised, to identify factors associated with the different ACE exposure profiles.

## Methods

### Study sample

We used data from Constances, a prospective cohort study of the French population, that includes 220 000 participants aged 18 to 69 at enrolment between 2012 and 2020.^21^ Eligible individuals were selected from the adult population affiliated to the French national health insurance system, which covers almost 85% of the population, using a random sampling scheme stratified on place of residence, age, sex, occupation and socioeconomic status to be representative of the general French population. Selected individuals were invited to visit one of the Constances examination centres for a health check-up as part of the first data collection wave. Individuals who gave their informed consent to participate in the study were included in the cohort. Participants are followed up annually by email or online questionnaires, depending on their preference, and undergo a medical examination every 4 years.

For this study, our sample consists of those volunteers who completed the 2020 follow-up questionnaire, the year in which questions on ACEs appeared in Constances. A flowchart of participant inclusion and a comparison of included vs excluded participants are provided in the online supplemental tables 6 and 7.

### Measures

#### ACE exposure

Childhood adversities were assessed using questions from the original version of ACE questionnaire used in the Behavioural Risk Factor Surveillance System (BRFSS).^22^ The questionnaire consisted of 16 self-reported items assessing various types of adversities experienced before the age of 18 years. These items covered several domains, including sexual violence, physical and emotional violence, household dysfunction, parental separation or divorce, parental death or severe illness, and family histories of incarceration, mental illness or substance use. A detailed description of all items and their response options is provided in online supplemental table 1. These items were translated into French specifically for use in the Constances cohort. The translation aimed to preserve the wording and content of the original BRFSS items as closely as possible. However, no formal psychometric validation of this French version has been conducted. We transformed the initial scale into three response categories: Yes (for once; more than once; or Yes), No (for Never; or No) and No answer (for I don’t know; I’m not sure or I prefer not to answer).

#### Covariables

Demographic factors potentially associated with ACE exposure were participants’ sex assessed via a self-reported item with response options ‘male’ and ‘female’, age at study inclusion (18–29, 30–39, 40–49, 50–59 and more than 60 years), and childhood socioeconomic characteristics including parents’ occupational grade (independent profession, intellectual profession, intermediate profession, employees, no profession or other) and parents’ geographical region of origin (France, Europe, Africa, Asia or Other) (online supplemental table 2).

### Statistical analyses

Descriptive statistical methods were used to describe the study populations’ demographic characteristics at the time of the study. For continuous variables, means and SD statistics were reported; frequencies and percentages were reported for categorical variables. Bivariate analyses were conducted using Pearson’s χ² tests or Fisher’s exact tests for categorical variables, and Wilcoxon rank-sum tests for continuous variables.

#### Latent class analysis

In this study, LCA was used to classify respondents with similar adversity profiles into distinct clusters based on their answers to 16 ACEs questions (online supplemental table 1: Q1 to Q16). Unlike approaches that rely on total ACE scores, LCA is a person-centred method that identifies unobserved subgroups with distinct patterns of exposure, allowing for a more nuanced understanding of risk.^23^ Within each class, a high probability of answering ‘yes’ to a specific question indicates that members in this class are more likely to be exposed to this particular type of ACE. This approach maximises homogeneity within sub-groups and heterogeneity between subgroups. To determine the optimal number of classes, the Bayesian Information Criterion (BIC, the Akaike Information Criterion (AIC), the maximum log-likelihood, the LMR (using the calc_lrt() function from the tidyLPA package, which allows computation of the LMR test based on model log-likelihoods, number of parameters, sample size and number of classes, as recommended by Bellemare-Lepage *et al*^24^) and entropy were compared.^25^ In the case of different solutions given by the fit statistics, we referred to the existing literature to find the best number of classes. Analyses were performed with the poLCA package of the statistical software R. Only participants with complete data on exposure to ACEs were included.

#### Multinomial logistic regression

To assess the association between ACEs class membership and demographic and childhood socioeconomic variables, multinomial logistic regression models were implemented. Independent variables included participants’ age, their parents’ socioprofessional category and parents’ region of origin. In this analysis, participants with missing values on any covariates were excluded. The proportion of missing data was low across covariates (ranging from 0% to 2%). Adjusted ORs were calculated with their CIs (95% CIs). All analyses were conducted separately to ensure sex-specific insights, with participants referred to as ‘female’ and ‘male’ in line with self-reported categories. All analyses were conducted using R Studio (V.4.4.1).

### Patient and public involvement

Patients and the public were not directly involved in the preparation of this manuscript, principally because the study used secondary analysis of existing data from a large, nationally representative cohort, which was not originally designed or implemented with patient or public involvement processes in place for individual studies using its data.

## Results

### Population description

Among study participants who responded to the 2020 follow-up Constances questionnaire, 101 071 completed all questions on ACEs. Table 1 provides a description of the study population by sex.

**Table 1:** Description by sex of sample and sociodemographic characteristics and prevalence of adverse childhood experience (ACE) categories of constances participants

Sample characteristics	Males, N=45 882*	Females, N=55 189*	
Age group (years)			
18–29	3878 (8%)	5834 (11%)	
30–39	8861 (19%)	11 270 (20%)	
40–49	10 813 (23%)	13 276 (24%)	
50–59	10 339 (22%)	12 415 (22%)	
+60	11 991 (26%)	12 394 (22%)	
Father’s socioprofessional category			
Intermediate profession	8106 (18%)	9215 (17%)	
Independent profession	8675 (19%)	11 508 (21%)	
Intellectual profession	10 053 (22%)	12 432 (23%)	
Employees	16 381 (36%)	18 451 (34%)	
No profession or other situation	1777 (3.9%)	2547 (4.7%)	
(Missing)	890	1036	
Mother’s socioprofessional category			
Intermediate profession	6516 (14%)	8264 (15%)	
Independent profession	5582 (12%)	7362 (14%)	
Intellectual profession	2859 (6.3%)	3860 (7.1%)	
Employees	13 306 (29%)	16 388 (30%)	
No profession or other situation	16 909 (37%)	18 523 (34%)	
(Missing)	710	792	
Father’s region of origin			
Mainland France	39 559 (87%)	47 741 (87%)	
Africa	2014 (4.4%)	1999 (3.6%)	
Asia	341 (0.7%)	493 (0.9%)	
Europe	3127 (6.9%)	3747 (6.8%)	
Other	553 (1.2%)	808 (1.5%)	
(Missing)	288	401	
Mother’s region of origin			
Mainland France	40 110 (88%)	48 474 (88%)	
Africa	1720 (3.8%)	1656 (3.0%)	
Asia	305 (0.7%)	472 (0.9%)	
Europe	3085 (6.8%)	3565 (6.5%)	
Other	402 (0.9%)	651 (1.2%)	
(Missing)	260	371	
Category of ACEs			P value†
Sexual violence	2820 (6.1%)	8889 (16.1%)	<0.001
Psychological violence	17 208 (37.5%)	20 589 (37.3%)	0.5
Physical violence	9874 (21.5%)	11 171 (20.2%)	<0.001
Parents’ separation	5410 (11.8%)	7363 (13.3%)	<0.001
Parents’ death	2554 (5.6%)	2980 (5.4%)	0.2
Parents/child Illness	9451 (20.6%)	14 349 (26.0%)	<0.001
Financial problem	9720 (21.2%)	14 114 (25.6%)	<0.001
Substance use	7448 (16.2%)	10 938 (19.8%)	<0.001
Violent environment	3930 (8.6%)	5301 (9.6%)	<0.001
Sentenced to prison	441 (1.0%)	646 (1.2%)	0.001
ACE score	1.69 (1.86)	2.05 (2.17)	<0.001

Percentages are based on available data. P values are shown only for ACE categories where sex-based comparisons were conducted.

* n (%); Mean (SD).

† Pearson’s χ2 test; Wilcoxon rank sum test.

In this sample, 54.6% were female participants and 45.3% were male participants. The majority of males were over 60 years at the time of the study, whereas most females were 40 to 49 years. Nearly 95% of study participants had French nationality at birth. The participants’ fathers were predominantly employees (office or commercial employee, child minder, manual worker, etc), while the mothers mostly had no profession or were in other situations. The most common region of origin was mainland France.

#### ACE prevalence

Table 1 shows the prevalence of ACE categories among study participants. Nearly 65% (n=69 406) reported experiencing at least one type of ACE. The most common ACE for both sexes was psychological violence.

For females, the second most frequently reported ACE categories were parental or child illness and financial problems, followed by physical violence and parental substance use. For the male sample, physical violence was the second most common category, followed by financial problems and parental or child illness. The largest sex difference was observed for the sexual violence category, with 16% of females reporting experiencing at least one type of sexual violence compared with 6% for males. The mean ACE score was 1.69 (SD=1.86) for males and 2.05 (SD=2.17) for females. More detailed information on individual ACEs and ACE scores is provided in online supplemental tables 3 and 4.

#### Latent classes description

**Figure 1:**
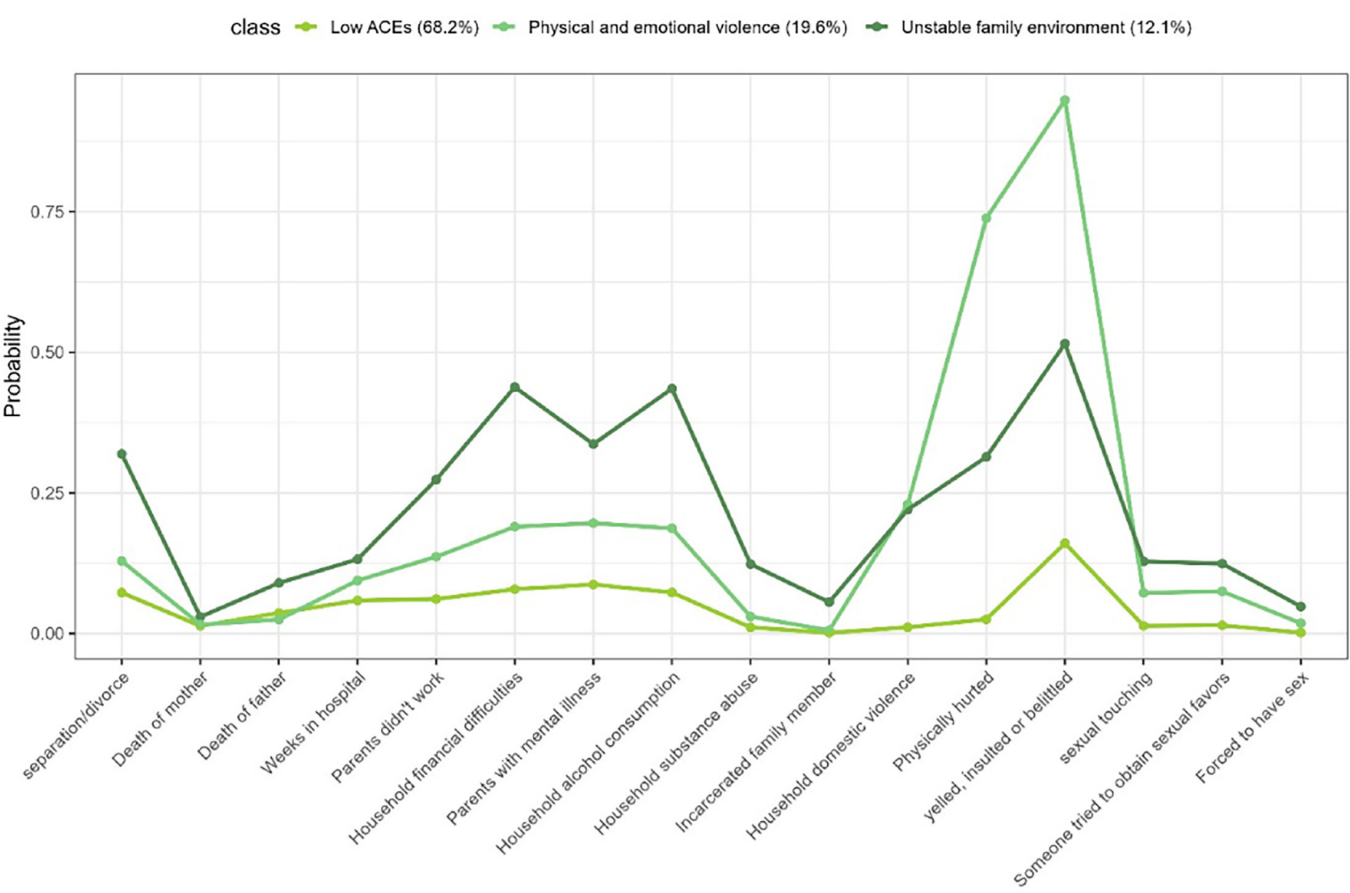
Profile plot based on latent class analysis for males. ACEs, adverse childhood experiences.

Figures1  2 illustrate groups identified by the LCA models for males and females respectively, alongside the probabilities of positive responses to the various items. Although statistical fit indices (AIC, BIC and entropy) were examined (online supplemental table 5), they did not converge to a single optimal model: AIC/BIC continued to decrease with additional classes, whereas entropy values were highest for the 3 and 4 class solutions. In line with the recommended LCA practice, we prioritised the interpretability and coherence of the class structure. The three-class solution for the male sample and the four-class solution for the female sample provided the best fit and most accurately described our data.

**Figure 2:**
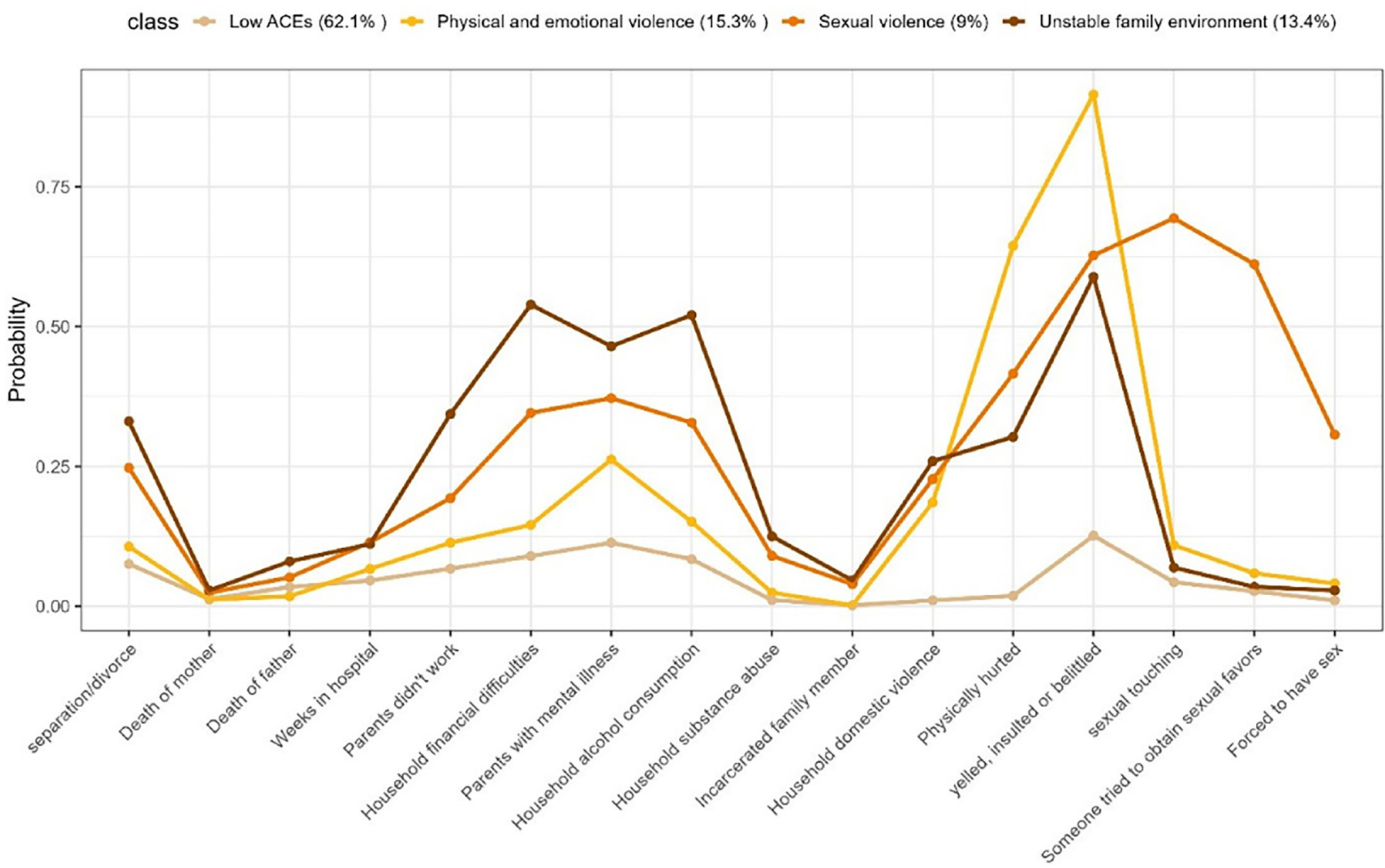
Profile plot based on latent class analysis for females. ACEs, adverse childhood experiences.

The ‘low exposure to ACEs’ class is characterised by participants with a low probability of exposure to adversities during childhood. This class represents the majority of participants, including 68.2% of males and 62.1% of females.

The ‘physical and emotional violence’ class includes individuals who reported being hit, physically injured, insulted or frequently belittled. This group comprises 15.3% of males and 19.6% of females.

The ‘unstable family environment’ class consists of participants who grew up in households with financial difficulties, violent conflicts between parents and parental alcohol and substance abuse. This class includes 13.4% of females and 12.1% of males.

For females, an additional ‘sexual violence’ group emerged, including those who experienced higher levels of exposure to sexual violence compared with other participants. Members of this group reported having been sexually assaulted or forced to have sex at least once (figure 2).

### Multinomial logistic regression

**Figure 3:**
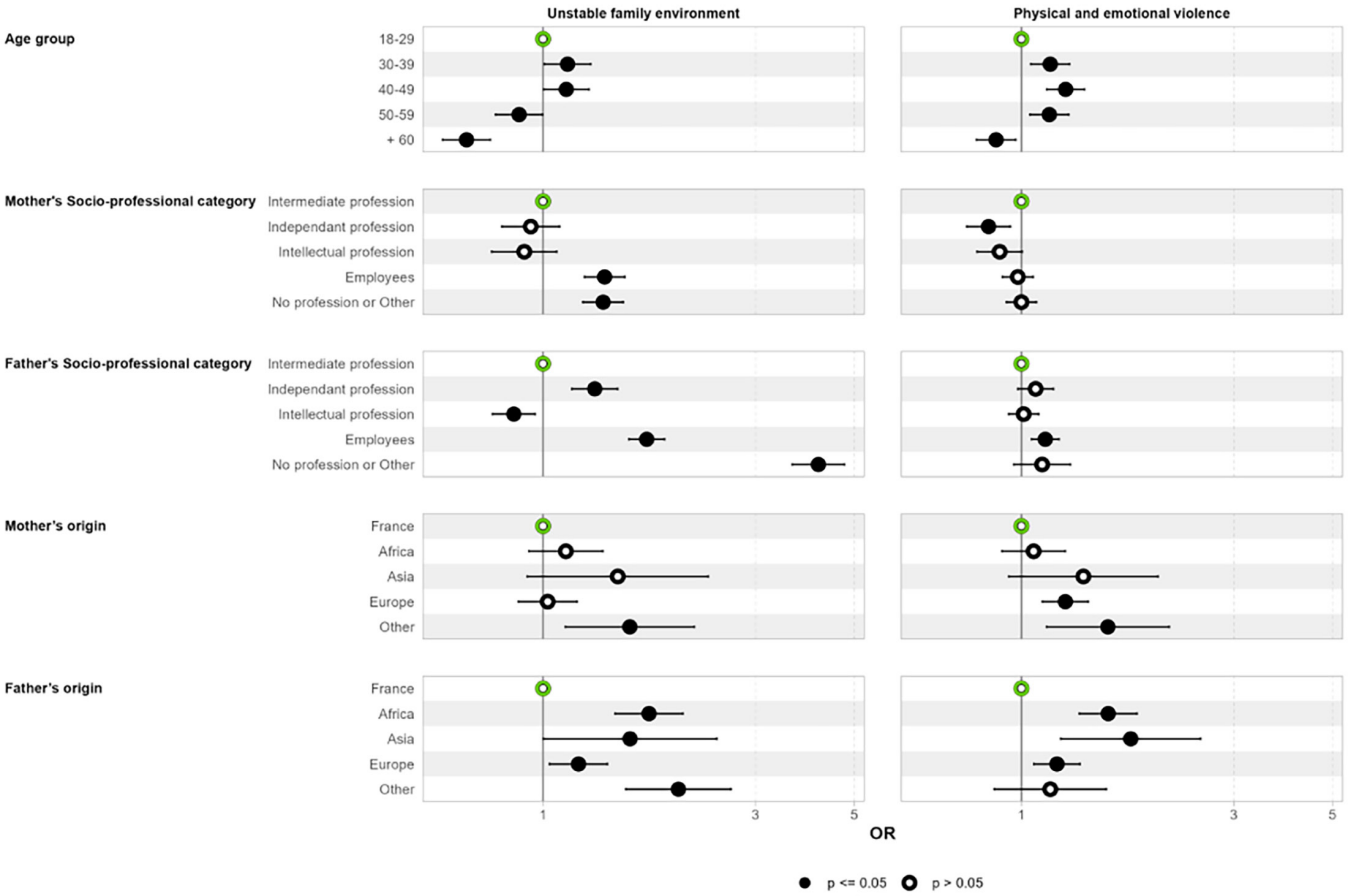
Associations between socioeconomic variables and identified latent classes among males. Forest plot illustrating the ORs and 95% CIs for the association between socioeconomic variables and membership of the different ACE classes for males. ACE, adverse childhood experience.

Figures3  4 show the adjusted associations between the LCA classes and demographic and socioeconomic variables for males and females respectively, using the ‘low ACEs’ group as the reference category.

**Figure 4:**
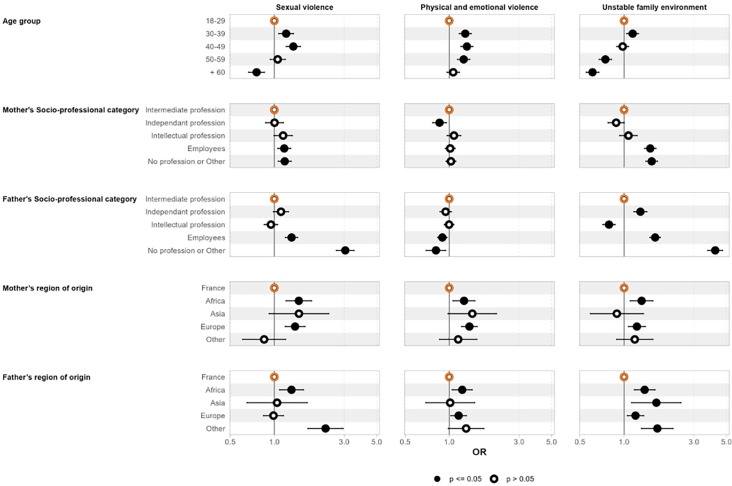
Associations between socioeconomic variables and identified latent classes among females. Forest plot illustrating the ORs and 95% CIs for the association between socioeconomic variables and membership of the different ACE classes for females. ACE, adverse childhood experience.

Individuals aged over 60 years had a reduced risk of exposure to ACEs compared with other age groups, while those aged 30–39 were most likely to be in the ‘unstable family environment’ and ‘physical and emotional violence’ classes. Regarding the ‘unstable family environment class’, all parental socioprofessional categories were associated with this class, except when the father had an ‘intellectual profession’.

In contrast, in the ‘physical and emotional violence’ class, males were less likely to belong to this class if their mothers were in an independent profession. However, having a father who was unemployed, an employee or in an independent profession significantly increased the odds of exposure to this type of violence. The mother’s region of origin, particularly when classified as ‘other’, was significantly associated with both classes. Additionally, participants were more likely to be in both classes compared with the ‘low ACEs’ class, when their fathers came from regions of Africa, Europe or other regions.

The results for females were quite similar to those observed in males. Significant associations were observed between demographic and childhood socioeconomic variables and LCA classes. Older age (over 60 years) was associated with significantly lower odds of belonging to the ‘sexual violence’ and ‘unstable family environment’ classes. Conversely, participants aged 30 to 39 had a significantly higher likelihood of being part of any of the three ACE classes, compared with the ‘low ACEs’ reference group.

With regard to parents’ socioprofessional category, females whose parents were not working or in other situations were significantly more likely to belong to the ‘unstable family environment’ class. The risk of being in the ‘sexual violence’ class was notably higher when the parents, and particularly the mother, had no occupation or when the participant preferred not to answer. Participants whose parents were born in Africa, Europe or other non-French regions had higher odds of belonging to any of the ACE classes compared with participants whose parents were born in mainland France.

## Discussion

This study explored the prevalence and distribution of ACEs among the French adult population. By using various methods of estimation, we were able to differentiate between types of childhood trauma and their frequency among males and females. With over 100 000 participants, this study provides one of the most comprehensive datasets available, addressing the gap in data on ACEs in France and establishing key reference points for future research. Our results showed that at least 70% of females and approximately 67% of males reported experiencing at least one traumatic event. Females were found to be more likely than males to be exposed to all categories of ACEs, with the exceptions of physical and psychological violence. LCA identified three distinct classes for males and four for females, each characterised by specific profiles of experiences. Additionally, the study revealed that age, parental socioprofessional category and geographical origin are associated with class membership. This comprehensive approach enhances our understanding of ACEs for different segments of the population. Although the questionnaire was administered in 2020 during the COVID-19 pandemic, there is no clear evidence from the literature that pandemic-related stress systematically distorted participants’ retrospective reporting of ACEs. On the other hand, retrospective self-reports of childhood maltreatment have been shown to be highly temporally stable, and current depressive symptomatology appears to influence them only marginally (eg, Goltermann *et al* found a small association between changes in depressive symptoms and changes in trauma recall.^26^

### Interpretation and comparison with the literature

The heterogeneity of ACE questionnaires, which are not standardised, combined with the varying characteristics of populations in different studies, makes comparisons with the wider literature challenging. Despite these differences, our findings are consistent with international literature. In our study, more than 65% reported at least one ACE, with psychological and physical violence most frequent, which is similar to patterns seen in North America and Asia, though European rates tend to be lower (emotional abuse: 6.2% for males, 12.9% for females, and physical abuse: 27.0% for males and 12.0% for females).^27^,^29^ The high prevalence observed in our sample may reflect increased awareness of childhood adversity in recent years, broader definitions of the items used in the questionnaire and the heterogeneous nature of the cohort.

Females reported a higher number of ACEs overall, consistent with previous studies.^30^,^32^ This is also reflected in the cumulative ACE scores, where females showed higher averages and a greater likelihood of having experienced multiple adversities. The higher prevalence of ACEs among women may be explained by both greater exposure to certain types of adversities such as sexual violence and a higher likelihood of sharing personal experiences, as societal norms can discourage men from reporting.^33 34^ Additionally, early caregiving roles often assumed by girls may heighten their awareness and reporting of ACEs.^35^ These findings underscore the need to account for gender differences in ACE research and highlight the value of stratified analyses to more accurately capture these patterns. They also suggest that the observed disparities may reflect gendered vulnerabilities, where social, cultural and structural factors place girls at greater risk for certain types of adversity.^36^ Recognising this gendered dimension helps to interpret the data more accurately, indicating that the higher prevalence of sexual and psychological violence among females may be less about individual characteristics and more about their socially constructed exposure to risk.

Beyond comparing ACE frequencies and cumulative scores, we used LCA to identify subgroups of individuals with similar patterns of adversity. LCA has been increasingly recommended in ACE research because it captures heterogeneity in exposure pathways that cumulative scores cannot.^37^ In our study, LCA was used to identify distinct profiles of adversity rather than assuming that all ACEs exert equal and additive effects.^20 38^ Although LCA offers a more nuanced understanding of ACE profiles, it has limitations, notably the subjectivity involved in determining the number of classes and interpreting them.^39^ In prior studies using LCA, researchers have typically identified between three and five distinct classes within the samples. Among the most identified classes are those with a low probability of exposure to ACEs (referred to as ‘low ACEs,’ ‘little ACEs,’ or ‘low adversity’) and a class characterised by participants with a high risk of exposure to multiple ACEs (‘high adversity,’ ‘multiple adversity,’ or ‘all ACEs’). Another frequently identified class includes individuals exposed to household dysfunction factors, such as domestic violence, financial difficulties, substance abuse, parental depression and illness (‘household dysfunction,’ ‘chaotic home’ or ‘family instability’). Finally, there is often a class associated with higher levels of physical abuse, emotional abuse and neglect (‘physical, sexual and emotional abuse,’ ‘child maltreatment’ or ‘physical and emotional abuse’). Our study differs from previous studies in the number and nature of identified classes. For example, Miedema *et al* identified an additional fifth class, named ‘Physical, sexual and emotional abuse,’ specifically among women. In our study, we found a distinct class involving sexual violence, but unlike Miedema *et al*, this class did not include other forms of abuse, such as physical and emotional violence.^20 40^ Even when the number of latent classes is similar to other studies, the composition and nature of adversity within these classes often differed by gender. For example, an American study also identified four classes in men and women, but the profiles diverged: in men, a class was defined by exposure to community violence, while in women, classes tended to reflect greater exposure to interpersonal forms of adversity, such as emotional and sexual abuse. This shows that similar class numbers can mask important gender variations in the type and severity of ACEs.^41^

Notably, few studies have stratified LCA results by sex. In our sample, males were more often assigned to the low ACEs class, while females were more often classified into higher adversity classes, including those characterised by sexual violence. We also found that participants over the age of 60 were less likely to be assigned to any of the higher adversity groups. This age-related trend may reflect generational differences in the recognition and reporting of childhood trauma. Older individuals may be less inclined to disclose negative experiences that were once considered taboo or stigmatised, whereas younger generations, supported by greater public awareness and more open discourse, may feel more comfortable reporting them. Additionally, shifts in societal norms, including increases in parental separation, mental health issues and substance use, may contribute to age-related differences in ACE exposure patterns.^42 43^

Our findings also support existing literature on the link between childhood socioeconomic variables and ACE exposure. Participants whose parents were unemployed or in low-status occupations were more likely to belong to classes such as ‘unstable family’ or ‘physical and emotional violence’. This is consistent with studies showing that parental unemployment significantly increases the risk of exposure to various types of adversity. A systematic review using data from 20 countries reported a 29% increase in the risk of sexual violence, a 54% increase in the risk of neglect, a 60% increase in the risk of physical violence, and a 90% increase in the risk of child maltreatment and parental mental illness when the parents are unemployed.^44 45^ Parental employment influences not only household income but also parental well-being, which in turn can impact parenting practices and family dynamics. Stress linked to economic insecurity can lead to greater irritability, strained parent-child interactions or even physical punishment and neglect.^46 47^

While few studies have focused on the role of parental geographical origin in ACE exposure, our results add nuance to understanding ACE prevalence across different contexts. Differing legal frameworks around child discipline and migration-related stressors experienced by some families may all influence ACE prevalence. For example, certain forms of physical punishment are still legal in certain countries but have been prohibited in France since 1990, which may impact how different populations perceive and report physical violence. Migrant families may also face socioeconomic difficulties, limited access to support systems and heightened stress, all of which can affect parenting behaviours and contribute to an unstable home environment.^48 49^ These findings underscore the need for culturally informed and context-sensitive approaches when investigating ACEs.

### Strengths and limitations

This is one of the first studies to assess ACE prevalence in the French adult population using a large, national cohort of over 100 000 participants. The sample size enhances the statistical power of our analyses and allows for detailed subgroup analyses. Additionally, the use of individual variables, cumulative ACE scores and LCA allows nuanced insights and comparisons with other studies. However, a major limitation lies in the reliance on a self-report questionnaire to measure ACEs, which may introduce the potential for recall bias, leading to either an underestimation or overestimation of exposure due to individual differences in perceptions of past events. Furthermore, although the questionnaire comprised 16 questions, it did not capture a broader range of negative experiences such as neglect, harassment or community violence, which can all significantly impact health outcomes. Although no formal validation of the French translation of the BRFSS ACE questionnaire was conducted, the translation closely preserves the content and response structure of the original instrument, which reduces the likelihood of substantial measurement discrepancies. Finally, the variables that were examined were limited to age, parental geographical origin and socioprofessional status. While the broader cohort includes data on variables such as place of birth and age at arrival in France, these were not available in the specific dataset used for this analysis. Including additional factors such as number of siblings, parental trauma history or individual migration experience would have provided a more comprehensive understanding of the individual early environment in which ACE exposure occurred. Another limitation relates to the demographic composition of the sample. More than 90% of participants were born in France, limiting our ability to examine ACE patterns among foreign-born individuals or recent immigrants. This is important because migration-related stressors, discrimination and socioeconomic precarity can shape both the exposure to and reporting of childhood adversity. Studies conducted in socially and economically vulnerable settings have shown that patterns of ACEs can differ substantially and may be strongly associated with later interpersonal violence and other adverse outcomes.^50^

## Conclusions

This study advances our understanding of childhood trauma within the French population, offering insights into the prevalence of the most common ACEs and the socioeconomic factors associated with them. Nevertheless, this topic warrants further exploration in France given the profound impact of childhood exposure to adverse experiences not only on health outcomes, but also on social behaviour, education and employment trajectories. Interventions targeting school, community and establishment welcoming children are crucial, particularly for girls and for children in socioeconomically fragile households. Strengthening early detection, trauma-informed education programmes and safe disclosure environments may help reduce long-term consequences. To more effectively guide public policy and interventions, further research is crucial to deepen our understanding of ACEs and their far-reaching consequences, thereby enabling decision-makers to develop more targeted and impactful measures.

## Supplementary material

10.1136/bmjph-2025-003300 online supplemental file 1

## Data Availability

Data may be obtained from a third party and are not publicly available.
