# Supplementary material for: Prevalence and profiles of adverse childhood experiences: a French nationwide study using the CONSTANCES cohort
Source: BMJ Public Health. 2026 Apr 9;4(2):e003300. doi: 10.1136/bmjph-2025-003300 (PMC13084877; doi:10.1136/bmjph-2025-003300)
Supplement: online supplemental file 1 [file bmjph-4-2-s001.docx]

**Supplementary tables**

| *Table S1: Description of the Adverse Childhood Experiences items in the Constances cohort* | | |
| --- | --- | --- |
|  | **Written questions** | **Response options** |
| *Q1* | *Before your 18th birthday, were your parents separate or divorced?* | Yes/No/I’m not involved/ I don’t know, I’m not sure |
| *Q2* | *Did your mother die before your 18's?* | Yes/No/ I’m not sure |
| *Q3* | *Did your father die before your 18's?* | Yes/No/ I’m not sure |
| *Q4* | *Prior to age 18: You spent several consecutive weeks in the hospital?* | Yes/No/ I’m not sure |
| *Q5* | *Prior to age 18: One of your parents didn’t work when he wanted to?* | Yes/No/ I’m not sure |
| *Q6* | *Prior to age 18: Your family had regular financial difficulties?* | Yes/No/ I’m not sure |
| *Q7* | *Prior to age 18: Did you live with someone who suffered from depression, mental illness, or was suicidal?* | Never /Once /More than once /I don't know, I'm not sure/I Prefer Not to Answer |
| *Q8* | *Prior to age 18: Did you live with someone who had excessive alcohol consumption or alcohol dependence?* | Never /Once /More than once /I don't know, I'm not sure/I Prefer Not to Answer |
| *Q9* | *Prior to age 18: Did you live with someone who was using illicit drugs or who was taking excessive amounts of medication (outside of a medical setting)?* | Never /Once /More than once /I don't know, I'm not sure/I Prefer Not to Answer |
| *Q10* | *Prior to age 18: Have you lived with someone who has been sentenced to prison (or other type of correctional facility) with or without a sentence arrangement?* | Never /Once /More than once /I don't know, I'm not sure/I Prefer Not to Answer |
| *Q11* | *Prior to age 18: How often did your parents or other adults at the home slap, punch or kick each other?* | Never /Once /More than once /I don't know, I'm not sure/I Prefer Not to Answer |
| *Q12* | *Prior to age 18: How often did a parent or other adult in the household hit you, beat you, kick you or physically hurt you?* | Never /Once /More than once /I don't know, I'm not sure/I Prefer Not to Answer |
| *Q13* | *Prior to age 18: How many times did a parent or other adult in the household yell at you, insult you or belittle you?* | Never /Once /More than once /I don't know, I'm not sure/I Prefer Not to Answer |
| *Q14* | *Prior to age 18: How many times did someone make you suffer sexual touching?* | Never /Once /More than once /I don't know, I'm not sure/I Prefer Not to Answer |
| *Q15* | *Prior to age 18: How many times did someone try to obtain from you, sexual favors?* | Never /Once /More than once /I don't know, I'm not sure/I Prefer Not to Answer |
| *Q16* | *Prior to age 18: How many times did someone force you to have sex?* | Never /Once /More than once /I don't know, I'm not sure/I Prefer Not to Answer |

| *Table S2: Description of the Socio-professional categories* | |
| --- | --- |
| Independent profession | Farmer, Craftsman, Shopkeeper, business owner |
| Intellectual profession | Executive, higher intellectual profession (engineer, physician, etc.) |
| Intermediate profession | Teacher, nurse, etc. |
| No profession or other | Without profession or in another situation |
| Employees | Office or commercial employee, child minder, manual worker, etc. |

| Table S3: Prevalence of individual Adverse Childhood Experiences by Sex (n (%)) | | |
| --- | --- | --- |
| **Adverse Childhood Experiences** | **Males** (N = 45 882) | **Females** (N = 55 189) |
| **Parents separation/divorce** |  |  |
| No | 39 853 (87%) | 47 096 (85%) |
| Yes | 5 410 (12%) | 7 363 (13%) |
| No answer | 619 (1.3%) | 730 (1.3%) |
| **Death of mother** |  |  |
| No | 45 103 (98%) | 54 286 (98%) |
| Yes | 748 (1.6%) | 884 (1.6%) |
| No answer | 31 (<0.1%) | 19 (<0.1%) |
| **Death of father** |  |  |
| No | 43 743 (95%) | 52 729 (96%) |
| Yes | 1 899 (4.1%) | 2 204 (4.0%) |
| No answer | 240 (0.5%) | 256 (0.5%) |
| **Consecutive weeks in the hospital** |  |  |
| No | 41 745 (91%) | 50 995 (92%) |
| Yes | 3 501 (7.6%) | 3 601 (6.5%) |
| No answer | 636 (1.4%) | 593 (1.1%) |
| **One of the parents didn’t work** |  |  |
| No | 37 959 (83%) | 44 694 (81%) |
| Yes | 4 862 (11%) | 6 989 (13%) |
| No answer | 3 061 (6.7%) | 3 506 (6.4%) |
| **Regular financial difficulties in the family** |  |  |
| No | 34 638 (75%) | 39 370 (71%) |
| Yes | 6 923 (15%) | 10 347 (19%) |
| No answer | 4 321 (9.4%) | 5 472 (9.9%) |
| **Lived with a parent with mental illness** |  |  |
| Never | 36 747 (80%) | 39 807 (72%) |
| Once | 6 146 (13%) | 10 853 (20%) |
| More than once | 460 (1.0%) | 916 (1.7%) |
| No answer | 2529 (5.5%) | 3613 (6.5%) |
| **Lived with someone who had excessive alcohol consumption** |  |  |
| Never | 37 706 (82%) | 43 455 (79%) |
| Once | 6 071 (13%) | 9 079 (16%) |
| More than once | 634 (1.4%) | 905 (1.6%) |
| No answer | 1471 (3.2%) | 1750 (3.2%) |
| **Household substance abuse** |  |  |
| Never | 43 874 (96%) | 52 435 (95%) |
| Once | 1 096 (2.4%) | 1 811 (3.3%) |
| More than once | 291 (0.6%) | 232 (0.4%) |
| No answer | 621 (1.4%) | 711 (1.3%) |
| **Incarcerated family member** |  |  |
| Never | 45 230 (99%) | 54 303 (98%) |
| Once | 403 (0.9%) | 615 (1.1%) |
| More than once | 38 (<0.1%) | 31 (<0.1%) |
| No answer | 211 (0.5%) | 240 (0.4%) |
| **Domestic violence between parents** |  |  |
| Never | 40 213 (88%) | 48 068 (87%) |
| Once | 1 473 (3.2%) | 1 866 (3.4%) |
| More than once | 2 457 (5.4%) | 3 435 (6.2%) |
| No answer | 1739 (3.8%) | 1820 (3.3%) |
| **An adult physically hurt you?** |  |  |
| Never | 34 203 (75%) | 42 188 (76%) |
| Once | 2 928 (6.4%) | 3 709 (6.7%) |
| More than once | 6 946 (15%) | 7 462 (14%) |
| No answer | 1805 (3.9%) | 1830 (3.3%) |
| **An adult yelled at you, insulted you, or belittled you?** |  |  |
| Never | 24 828 (54%) | 30 681 (56%) |
| Once | 2 693 (5.9%) | 3 325 (6.0%) |
| More than once | 14 515 (32%) | 17 264 (31%) |
| No answer | 3846 (8.4%) | 3919 (7.1%) |
| **Someone made you suffer sexual touching** |  |  |
| Never | 43 433 (95%) | 47 042 (85%) |
| Once | 1 119 (2.4%) | 3 176 (5.8%) |
| More than once | 793 (1.7%) | 3 294 (6.0%) |
| No answer | 537 (1.2%) | 1677 (3.0%) |
| **Someone tried to obtain sexual favors** |  |  |
| Never | 43 466 (95%) | 48 862 (89%) |
| Once | 1 247 (2.7%) | 2 565 (4.6%) |
| More than once | 692 (1.5%) | 2 219 (4.0%) |
| No answer | 477 (1.0%) | 1543 (2.8%) |
| **Forced to have sex** |  |  |
| Never | 45 030 (98%) | 51 510 (93%) |
| Once | 268 (0.6%) | 1 391 (2.5%) |
| More than once | 253 (0.6%) | 1 105 (2.0%) |
| No answer | 331 (0.7%) | 1183 (2.1%) |
|  | | |

| *Table S4: Adverse Childhood Experiences Cumulative Scores (N (%))* | | |
| --- | --- | --- |
|  | **Males, N = 45 882** | **Females, N = 55 189** |
| **ACE Score** |  |  |
| 0 ACE | 15 360 (33.4%) | 16 305 (29.5%) |
| 1 ACEs | 10694 (23.3%) | 11889 (21.5%) |
| 2 - 3 ACEs | 12828 (27.8) | 15228 (27.4%) |
| 4 or more ACEs | 7 000 (15.5%) | 11 767 (21.6%) |
|  | | |

| *Table S5: Latent Class Model Selection Statistics* | | | | | | | | |
| --- | --- | --- | --- | --- | --- | --- | --- | --- |
|  | **Males** | | | | **Females** | | | |
| Classes | AIC | BIC | Entropy | Maximum log-likelihood | AIC | BIC | Entropy | Maximum log-likelihood |
| 2 classes | 554533.1 | 555100.8 | 0.72 | -277201.6 | 751452.2 | 752031.9 | 0.73 | -375661.1 |
| 3 classes | 545442.9 | 545442.9 | 0.77 | -272623.4 | 738441.6 | 739315.6 | 0.80 | -369122.8 |
| 4 classes | 538587 | 539731.2 | 0.81 | -269162.5 | 731512.5 | 732680.9 | 0.77 | -365625.3 |
| 5 classes | 534223.3 | 535655.7 | 0.81 | -266947.7 | 725610.5 | 727073.1 | 0.76 | -362641.2 |

*Table S6 : Flowchart of participants*

*
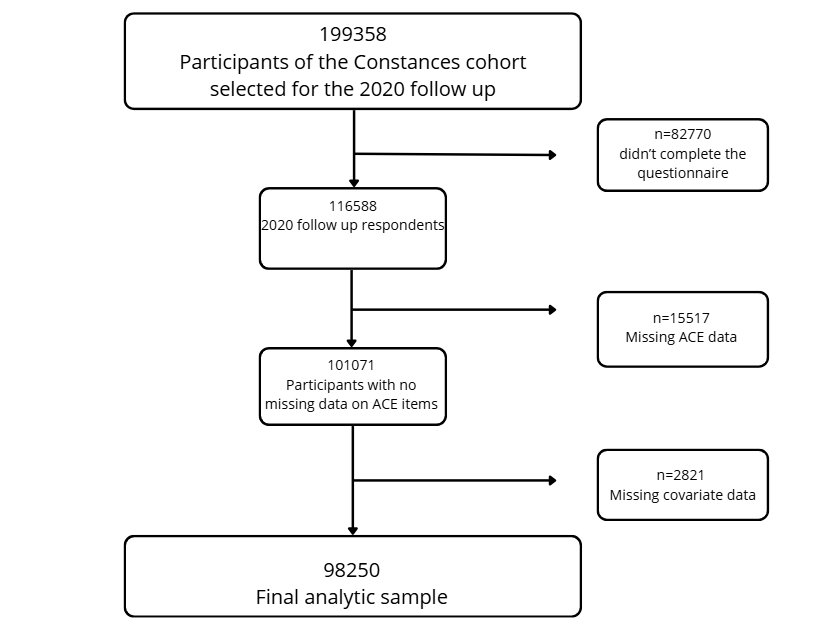
*

*Table S7: Comparison of the excluded and included participants*

|  | excluded, N = 15,517 | included, N = 101,071 | p-value |
| --- | --- | --- | --- |
| Age groups (years) |  |  | <0.001 |
| 18-29 | 731 (4.7%) | 9712 (9.6%) |  |
| 30-39 | 1788 (12%) | 20131 (20%) |  |
| 40-49 | 3054 (20%) | 24089 (24%) |  |
| 50-59 | 3960 (26%) | 22754 (23%) |  |
| + 60 | 5984 (39%) | 24385 (24%) |  |
| Participant's Sex |  |  | <0.001 |
| male | 6752 (44%) | 45882 (45%) |  |
| female | 8765 (56%) | 55189 (55%) |  |
| Mother’s region of origin |  |  | <0.001 |
| Mainland France | 13130 (86%) | 88584 (88%) |  |
| Africa | 711 (4.7%) | 3376 (3.4%) |  |
| Asia | 124 (0.8%) | 777 (0.8%) |  |
| Europe | 1087 (7.1%) | 6650 (6.6%) |  |
| Other | 194 (1.3%) | 1053 (1.0%) |  |
| Father’s region of origin |  |  | <0.001 |
| Mainland France | 12840 (84%) | 87300 (87%) |  |
| Africa | 810 (5.3%) | 4013 (4.0%) |  |
| Asia | 123 (0.8%) | 834 (0.8%) |  |
| Europe | 1130 (7.4%) | 6874 (6.8%) |  |
| Other | 330 (2.2%) | 1361 (1.4%) |  |
| Mother’s Socio-professional category |  |  | <0.001 |
| Intermediate profession | 1639 (11%) | 14780 (15%) |  |
| Independant profession | 2373 (16%) | 12944 (13%) |  |
| Intellectual profession | 677 (4.5%) | 6719 (6.7%) |  |
| Employees | 4257 (28%) | 29694 (30%) |  |
| No profession or Other | 6126 (41%) | 35432 (36%) |  |
| Father’s Socio-professional category |  |  | <0.001 |
| Intermediate profession | 2163 (14%) | 17321 (17%) |  |
| Independant profession | 3372 (23%) | 20183 (20%) |  |
| Intellectual profession | 2614 (17%) | 22485 (23%) |  |
| Employees | 5759 (39%) | 34832 (35%) |  |
| No profession or Other | 1033 (6.9%) | 4324 (4.4%) |  |
| Participant's Nationality |  |  | <0.001 |
| French by birth | 14151 (93%) | 94788 (95%) |  |
| Foreign national | 417 (2.7%) | 2125 (2.1%) |  |
| Naturalised French | 630 (4.1%) | 3265 (3.3%) |  |
| Participant’s Socio-professional category | |  | <0.001 |
| Intermediate profession | 4313 (30%) | 29848 (31%) |  |
| Independant profession | 329 (2.3%) | 1856 (1.9%) |  |
| Intellectual profession | 3972 (28%) | 34026 (35%) |  |
| Employees | 5010 (35%) | 26255 (27%) |  |
| No profession or Other | 711 (5.0%) | 4474 (4.6%) |  |
